# Tennessee State Medical Society

**Published:** 1874-05

**Authors:** 


### TENNESSEE STATE MEDICAL SOCIETY.

Through the courtesy of Dr. W. B. Wells, of Red Clay, Ga., we are enabled to present the following abstract of the proceedings of this society, culled from the reports of the Chattanooga *Daily Times*, and the Chattanooga *Daily Commercial.*

The Forty-first Annual Session of this body convened in Chattanooga, on Tuesday, the 7th April. Dr. Thos. Lipscomb in the chair, in the absence of the President, Dr. J. J. Abernathy. After organization and the election of new members, Dr. J. B. Murfree, of Murfreesboro, was elected President for the ensuing year, and Dr. D. Y. Green, of Chattanooga, Dr. J. T. Evans, of Union City, and Dr. T. B. Buchanan, of Nashville, Vice Presidents.

The following essays were read: *Diet of Parturient Women*, by Dr. J. J. Abernathy, of Decherd; *Diptheria and Malignant Sore Throat*, by Dr. J. B. Murfree, of Murfreesboro; *Necessity of a State Board of Health*, by Dr. E. M. Wright; *Puerpereal Peritonitis*, by Dr. Thomas Lipscomb, of Shelbyville, who referred to the fact that it was now forty-three years since he first attended a meeting of this society, and gave some interesting reminiscences of the old members of the society. He related the particulars of the treatment of three cases of puerperal peritonitis and one of acute non-puerperal peritonitis, by sulphate of morphia in *one grain* doses every three hours in conjunction with veratrum viride. For the latter remedy he claimed specific powers for the control of this disease, and also in all fevers, excepting those of malarial origin, and urged the younger members of the profession to adopt its use in their practice. Dr. Abernathy inquired if he weighed the doses of morphine, to which Dr. Lipscomb replied in the affirmative. Dr. Sims suggested that morphia and veratrum were antagonistic to each other, and the administration of the one rendered the patient more tolerant of the other. Dr. W. T. Briggs agreed with what had been said, and remarked that the disease was always caused by septic poisoning, and the best preventive is cleanliness.

Drs. Lipscomb, Lindsley and Lenoir, committee appointed to negotiate with railroads, presented the following resolutions for adoption:

*Resolved,* That the Tennessee Medical Society does not meet for base or sordid purposes, but for the promotion of science, and in the interests of humanity.

2. *Resolved,* That as we thus devote our time and expend money for these objects, all reasonable favors should be extended to us.

3. *Resolved,* That having so generally received courtesy and kindness from railroads which have usually acted with their characteristic liberality towards us, we have been pained no less than surprised at an entirely different course by some of our roads.

4. *Resolved,* That the following roads have obtained this unenviable notoriety: East Tennessee, Virginia & Georgia Railroad, and the Memphis & Charleston Railroad.

5. *Resolved,* That this refusal to respond to the claims of science and the cause of humanity is not the ordinary course of railroads, and should receive the decided disapprobation of every citizen.

6. *Resolved,* That the foregoing resolutions be published to the world as the sense of the Tennessee Medical Society.

On motion of Dr. Sims, a resolution was adopted favoring the Tennessee Medical Colleges.

The President, Dr. Abernathy, read his address to the Society:

He was convinced that the dignity, energy and virtue of the profession were well represented in the Society—that its work was a great and important one, and would continue to be a zealous and laborious struggle in the pursuit of truth and the facts of science. The profession had always been embarrassed by a set of camp-followers and pretenders who do incalculable mischief by the spread of deleterious and pernicious nostrums. He advocated the passage of a law to prevent the sale of nostrums, and to regulate the conferring of diplomas; also, to establish a home for the cure of drunkenness and the opium habit, which prevailed so extensively. He did not think it necessary to give alcoholic mixtures for diseases, and many times the use of alcohol was pernicious in its effects. He referred to the old methods of treating diseases, and to the wonderful, almost startling improvements that have been made in surgery and physic. He predicted that the time would come when medical science will have advanced so far as almost to prevent disease.

An essay sent by Dr. Paul Eve, on Esmarch's *Bloodless Operation on the Extremities,* was read, describing amputation of the thigh, removal of necrosis of the tibia, treatment of gunshot wounds, etc., by the application of the rubber bandage or elastic tourniquet. This method has but recently been suggested and yet it is being everywhere resorted to by the most eminent of the profession. The conviction is becoming general that Esmarch's Bloodless Operation is a decided success, and marks an advance step in practical surgery.

Dr. Briggs remarked that it was not always necessary to perform a bloodless operation, and in some cases it would be positively injurious, as in persons of a full habit, and those who had not suffered from exhaustion.

An essay on the *Philosophy of the New Chemistry* was next read by Dr. A. S. Wiltse. The paper went exhaustively into the definition of chemical terms, and treated upon the reduction of matter to original elements and the effects of various combinations of matter.

Dr. J. S. Burn next read a very fine production on *Hypodermic Injection of Narcotics,* comparing it with the usual method of administering morphia, quinia, etc. He gave in detail the experience of his practice, and was fully convinced that this method of allaying pain was safe, easy and reliable. In the treatment of neuralgia and cholera previous to collapse, he had been very successful in the use of hypodermic injections.

The essay of Dr. Somers, on *Digestion,* was read in his absence by Dr. Buchanan. It began by reference to the discovery of the gastric juice and its action. The function of the gastric juice was the digestion of nitrogenous matter, and when there is a deficiency of acid in the juice, much of this matter is not digested, and hence the necessity of appropriate material to furnish the proper amount of acid. The paper contained some good suggestions on diet, and recommended a moderate use of water at meals.

On motion the society adjourned, to meet at Nashville, on the first Tuesday of April, 1875.

